# The Need for Early Detection and Treatment in Alzheimer's Disease

**DOI:** 10.1016/j.ebiom.2016.07.001

**Published:** 2016-07-05

Despite its description over 100 years ago by German psychiatrist Alois Alzheimer, there has been little effective translation of insights on Alzheimer's disease (AD) pathobiology into effective treatments. As of 2015, roughly 50 million people globally — most over the age of 65 years — have dementia, with AD accounting for 60–70% of cases. Already, AD is the sixth leading cause of death in the US and the leading killer of women in the UK. As the population ages, AD is expected to affect 135 million people by 2050. Within the formal healthcare system in the US, AD currently costs USD 236 billion per year, a number that does not account for over 18 billion unpaid hours by those family and friends voluntarily caring for AD patients. With June 2016, Alzheimer's Awareness Month, just behind us and the Alzheimer's Association International Conference convening July 24–28, 2016, now seems a good time to discuss the looming crisis of AD.

AD patients initially present with mild cognitive impairment (MCI): occasional forgetting of details, misplacing of items, and other symptoms that may be attributed to other factors like stress or simply “aging”. Early-stage AD brings more noticeable memory deficits, such as forgetting familiar names and events, and confusion in unfamiliar situations. Ultimately, late-stage AD patients may be non-verbal or incoherent, have severe sleep and motor deficits, and become increasingly aggressive, paranoid, or unresponsive. An emerging, frightening theme from recent studies is that by the time cognitive impairment is noticed by a patient, their close relations, or doctor, the cascade of events leading to full-blown AD may be irreversible without a disease-modifying therapy. At present, only five drugs are US FDA-approved for AD. None are disease-modifying but instead are intended for symptom management, with varying degrees of efficacy.

It is clear that AD biomarkers are critical for catching the disease early to allow for preventative interventions. The only definitive genetic biomarkers for AD are mutations in amyloid precursor protein (*APP*) or the presenilin enzymes 1 and 2 (*PSEN1* and *PSEN2*). Each of these genes contribute to the aberrant production of the peptide Aβ40/42, which aggregates into the amyloid plaque deposits in the brain that are a signature of AD. *APP* and *PSEN* mutations, though, only account for a small fraction, roughly 5%, of early-onset familial AD. A vast majority of AD cases are late-onset and sporadic. Large-scale genome-wide association studies (GWAS) are ongoing to identify risk and protective factors for AD. Hundreds of genes have already been implicated, including the most significant *APOEε4* variant, which confers a 5 to 15-fold higher risk for developing AD. As more GWAS hits emerge, it is the hope that they may reveal other reliable mono- or oligogenic signatures of increased risk for AD that will allow patients to enroll in early interventions.

Non-genetic AD biomarkers are also being extensively explored. It has been shown that a decade or more before MCI onset, increased Aβ42 levels can be detected in patients' cerebrospinal fluid (CSF). Subsequent to Aβ42 detection in CSF, positron emission tomography (PET) imaging can identify amyloid deposition in the brain. But amyloid is not the only culprit in AD pathology. Among other emerging putative causes of AD — ranging from inflammation to metabolic dysfunction — tau is another well-studied AD hallmark. This microtubule-associated protein becomes hyperphosphorylated and aggregates into neurofibrillary tangles in AD patients' brains. Elevated tau levels are also seen in patients' CSF long before MCI onset, and recent advances in tau PET tracers now allow detection of total or misfolded tau in the brain. CSF sampling and PET imaging are not inexpensive, routine, or comfortable procedures, however, and indeed their predictive power is still somewhat controversial. These factors have led to a growing field screening for serum markers for pre-AD. While it is still early days, proof-of-concept studies showing elevated neurofilament levels in pre-AD patients or the presence of Aβ42 autoantibodies are paving the way for future blood-based diagnostics for early AD intervention.

There is no shortage of investigational AD drugs. However, between 2000 and 2012, while over 400 AD clinical trials were undertaken, a recent study suggested that 99.6% of them ended in failure (compared to anti-cancer drugs, with a roughly 80% failure rate). One reason for such abysmal results may be that studies were initiated too late in disease, after the cascade toward AD has become inevitable. Herein lies the promise for pre-AD biomarkers. Several ongoing clinical trials aim to prevent AD before the disease can take root. One study is investigating the amyloid-targeting antibody solanezumab in the Anti-Amyloid Treatment in Asymptomatic Alzheimer's Disease (A4) trial, where patients already have appearance of amyloid plaques but are cognitively normal, hoping that removing plaques before the disease progresses will be beneficial. In another study (Generation trial), only patients homozygous for the *APOEε4* variant, who are asymptomatic but at increased risk for developing AD, are being recruited for treatment with either an investigational Aβ plaque-clearing immunotherapy or an inhibitor targeting Aβ production. Because of the protracted natural history of AD and relatively soft clinical end points (like cognitive ability and day-to-day functional activity), these trials are often exceedingly long, costly, and difficult to interpret, requiring considerable resolve from all stakeholders involved.

This issue of *EBioMedicine* includes a research article by Letronne et al. on a human protein, ADAM30, which may affect amyloid deposition in the brain but has not been well-studied because it is not normally expressed in the mouse, a common AD model organism. The accompanying commentary by Becker-Pauly and Pietrzik highlights the need for alternative approaches to modeling AD to maximize the likelihood for translation to the clinic. Likewise, ingenuitive approaches to discovering druggable targets, to validating biomarkers for early diagnosis and as surrogate end points, and to optimizing clinical trials for investigational AD therapies are desperately needed. Only when all of these factors coalesce will we begin to effectively solve the enigma of AD and change the lives of those hundreds of millions of people directly and indirectly affected by this tragic disease.

-*EBioMedicine*

